# Renal outcomes in pediatric patients with sickle cell disease: a single center experience in Saudi Arabia

**DOI:** 10.3389/fped.2023.1295883

**Published:** 2023-12-15

**Authors:** Dania A. Monagel, Shatha S. Algahtani, Lian A. Karawagh, Wafa D. Althubaity, Sara A. Azab, Deena F. Haneef, Naglla Elimam

**Affiliations:** ^1^ College of Medicine, King Saud bin Abdul-Aziz University for Health Sciences, Jeddah, Saudi Arabia; ^2^ King Abdullah International Medical Research Center, Jeddah, Saudi Arabia; ^3^ Department of Oncology, Ministry of the National Guard-Health Affairs, Jeddah, Saudi Arabia; ^4^ Center of Excellence in Genomic Medicine Research (CEGMR), King Abdulaziz University, Jeddah, Saudi Arabia

**Keywords:** sickle cell disease, pediatric, renal, complications, nephropathy

## Abstract

**Background:**

Sickle cell nephropathy (SCN) is a significant complication of sickle cell disease (SCD) with an asymptomatic onset in childhood and potential progression to chronic kidney disease (CKD). The clinical findings of SCN include hyposthenuria, hematuria, proteinuria, hyperfiltration, and CKD. Data on renal manifestation among patients with SCD in Saudi Arabia is lacking. Therefore, this study aimed to evaluate renal outcomes in patients with SCD who visited a hematology clinic at the National Guard Hospital, Jeddah.

**Methods:**

We conducted a retrospective chart review of renal complications in patients with SCD who are within 0–14 years of age and on regular follow-ups at the pediatric hematology clinic in King Abdulaziz Medical City-Jeddah, Saudi Arabia.

**Results:**

Among the 140 patients with SCD, 99 met the inclusion criteria. The median age at diagnosis was 18 (1–108) months. Two SCD phenotypes were observed, with 82 (83%) patients having sickle cell anemia (HbSS) and 17 (17%) having HbS/B^+^ thalassemia. Of the total patients, 92 (93%) were administered hydroxyurea (HU), with a median starting age of 48 (9–168) months. The most common renal complication observed during routine urinalysis was hematuria (38%), followed by proteinuria (11%). After stratifying the sample into four age groups (0–3 years old, 4–7 years old, 8–11 years old, and 12–14 years old), the mean glomerular filtration rate (GFR) values were 96.16, 101.36, 112.69, and 120.11 ml/min/1.73 m^2^ respectively. Renal imaging revealed abnormal findings in 27 (29%) patients. The most common abnormality observed on US was increased echogenicity (43%).

**Conclusion:**

SCN is a significant complication of SCD. In this study, we assessed the renal outcomes in pediatric patients with SCD. After analyzing the clinical findings of SCN, we concluded that the presence of renal complications in pediatric patients presented a progressive pattern.

## Introduction

Sickle cell disease (SCD) is an autosomal recessive disorder characterized by a missense mutation in the beta globin-6 that leads to hemoglobin S production (1). The clinical manifestations of SCD are unpredictable, and the disease is often associated with substantial morbidity and mortality (2). The hallmark of SCD is vaso-occlusion followed by chronic hemolysis, which usually has a multisystem impact (3).

The renal complications of SCD include hyposthenuria (low specific gravity urine), hematuria, albuminuria/proteinuria, glomerular hyperfiltration [supraphysiological elevation in the glomerular filtration rate (GFR)], and chronic kidney disease (CKD) (4). Sickle cell nephropathy (SCN) is a significant overlooked complication of SCD, with asymptomatic onset in childhood and possible progression to CKD by late adolescence or early adulthood (5). The risk factors for progression into CKD are not well defined; however, significant albuminuria, the most common presentation in childhood, is a critical factor in progression (6). Albuminuria in SCD is caused by endothelial activation and inflammation due to sickled red blood cells, which induce hemolysis following the release of hemoglobin (7, 8).

Other kidney injuries such as hyposthenuria occur because of renal medullary sickling, which destroys the vasa recta (9). Another abnormality is hematuria, caused by increased pressure in the left renal vein due to compression by the aorta and superior mesenteric artery (7, 10–12). Hyperfiltration, defined as estimated GFR (eGFR) >180 ml/min/1.73 m^2^, is common and occurs early in children with SCD. As children age, the GFR rises further and can peak at levels above 200 ml/min per 1.73 m^2^. This may be partially compensated by an increase in cardiac output due to anemia (13, 14).

It is generally agreed that optimal management of individuals with SCD requires comprehensive multidisciplinary care (15, 16). Routine monitoring for early signs of renal impairment and early start of hydroxyurea as a disease-modifying therapy are advised as preventative measures for late-stage renal disease (17). Providers should bear in mind that serum creatinine as a marker of glomerular function in these patients has its limitations. Therefore, the pattern and rate of change of either serum creatinine or eGFR should be considered instead of the absolute value. In particular, comparatively modest changes in creatinine at higher levels of GFR may represent a vital decline in renal function (13, 14, 17, 18). Once SCN is diagnosed, several supportive care measures can be utilized. These include the use of angiotensin-converting enzyme (ACEi) or angiotensin II receptor blockers (ARBs), early initiation of hydroxyurea (HU) therapy although it is standard of care now, treatment for hypertension and involvement of a nephrologist are appropriate (17). Further progression to CKD may warrant exchange transfusions and renal transplantation (17).

The long-term effects of SCD on renal function among patients with SCD in Saudi Arabia remain obscure. Therefore, this study aimed to assess renal outcomes among patients with SCD who visited a pediatric hematology clinic at the National Guard Hospital, Jeddah. This will likely help in establishing specific guidelines to monitor renal function in this unique population and promptly provide necessary interventions.

## Methods

This descriptive retrospective cohort study reviewed the medical charts of all children with SCD from 2016 to 2020. This study was conducted at a pediatric hematology clinic in King Abdulaziz Medical City (KAMC) in Jeddah, Saudi Arabia. The study included patients with SCD aged 0–14 years and were regularly followed up at the pediatric hematology clinic. Patients with sickle cell traits and those lost to follow-up were excluded from the study. The Institutional Review Board of the King Abdullah International Research Center approved the study and waived the requirement for informed consent due to the retrospective nature of the study.

The total number of patients population at the National Guard Hospital in Jeddah was 140; after applying the study inclusion criteria, 99 patients were included in this study. Electronic medical records from the BestCare system: Health records were obtained per Case Report Form, and the collected data were organized. The data included the patient's demographics, medical history, renal complications, treatments, outpatient visits, inpatient admissions, and radiological investigations, mainly ultrasound. Notably, most patients who attended the clinic underwent abdominal ultrasound as part of routine care to assess the liver, spleen, gallbladder and kidney.

Serum creatinine levels were used to calculate GFR. Although monitoring renal function using serum creatinine levels is simple and practical, it is usually inaccurate in children due to factors such as age, sex, height, and muscle mass (19). GFR was calculated using the Bedside Schwarz Pediatric Calculator (20). The GFR data were stratified into four age groups (0–3 years old, 4–7 years old, 8–11 years old, and 12–14 years old) for graph plotting. GFR <60 ml/min/1.73 m^2^ was considered as glomerular dysfunction, while a GFR >150 ml/min/1.73 m^2^ was considered as glomerular hyperfiltration. The dosages of hydroxyurea administered were set in the ranges (>30 mg/kg, 25–30 mg/kg, 20–24.9 mg/kg, 15–19.9 mg/kg, and <15 mg/kg). The total number of crises, including vaso-occlusive crises (VOC), acute chest syndrome (ACS), and hyperhemolytic crises, was also stratified into ranges before the analysis.

### Statistical analysis

JMP software (John's Macintosh Project), version 10.0 (SAS Institute Inc., Cary, NC, USA) was used to analyze the collected data. Numerical data are described as mean [standard deviation (SD)], median, maximum, and minimum. Categorical data are presented as numbers and frequencies (%). Chi-square test and one-way analysis of variance (ANOVA) were used to analyze the associations between categorical variables. Simple logistic regression test was used to analyze the associations between categorical and continuous variables. Statistical significance was set at *P* < 0.05.

## Results

### Patient demographics

Among 140 patients with SCD, only 99 (70.7%) met the inclusion criteria. Of the 99 patients, 50 (50.5%) were females, and 49 (49.5%) were males as shown in Table 1. The majority, 82 (82.8%) had sickle cell anemia (HbSS), and only 17 (17.2%) had HbS/B^+^ thalassemia. Of the 82 patients with sickle cell disease, 46 (56.1%) had high HbF levels. Comparison of the risk of renal complications among the different SCD genotypes revealed no significant results.

**Table 1:** Baseline patients’ characteristics.

	*N* = 99	%
Gender		
Male	49	49.4
Female	50	50.1
Median Age at diagnosis in (months)	18 (1–108)	
SCD Phenotypes		
HbSS	82	83
HbS/beta thalassemia	17	17
Sickle cell with high HbF (HbSS patients only)		
Yes	46	56
No	36	44
HU usage		
Yes	92	93
No	7	7
HU median starting age (months)	48 (9–168)	
HU dosage (mg/kg)		
>30	20	22
25–30	23	25
20–24.9	18	20
15–19.9	20	22
<15	11	12
Median HU Duration (months)	36 (1–144)	
Blood transfusion		
Yes	65	66
No	34	34
Deferasirox usage		
Yes	5	5
No	94	95
NSAIDs usage		
Yes	54	55
No	45	45
Vancomycin usage		
Yes	52	53
No	47	47
Aminoglycoside usage		
Yes	1	1
No	98	99

The median age (range) at diagnosis was 18 (1–108) months, with a mean of 31.5 months (SD = 30). None of the patients underwent nephrectomy, and only one had a congenital renal anomaly. One patient had nocturnal enuresis and two (2.02%) reported urinary tract infection (UTI). Painful VOC was the most frequent crisis, occurring in 49 (49.5%) patients, followed by ACS in 39 (39.4%), and hyperhemolytic crises in 17 (17.17%). After stratifying the number of crises into ranges, 43 (66.2%) had <5 crises, followed by 6–10 crises in 11 (16.2%) patients. Simple logistic regression test revealed that the association between the number of crises per patient and renal complications (proteinuria, hyperfiltration, glomerular dysfunction, and UTI) was statistically significant (*P* < 0.0001).

### Renal outcome

**Figure 1:**
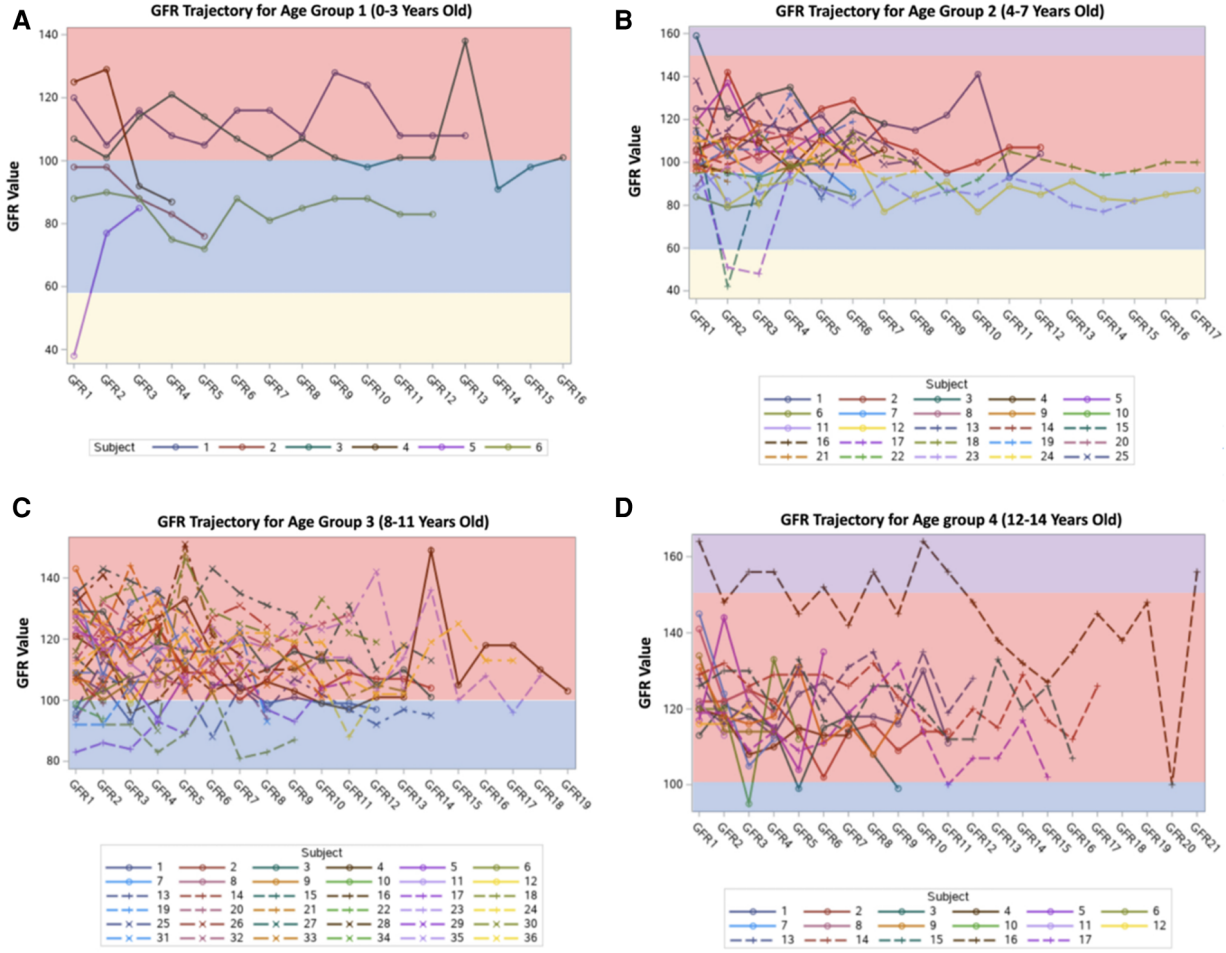
GFR trends among patients (multiple time points).

**Figure 2:**
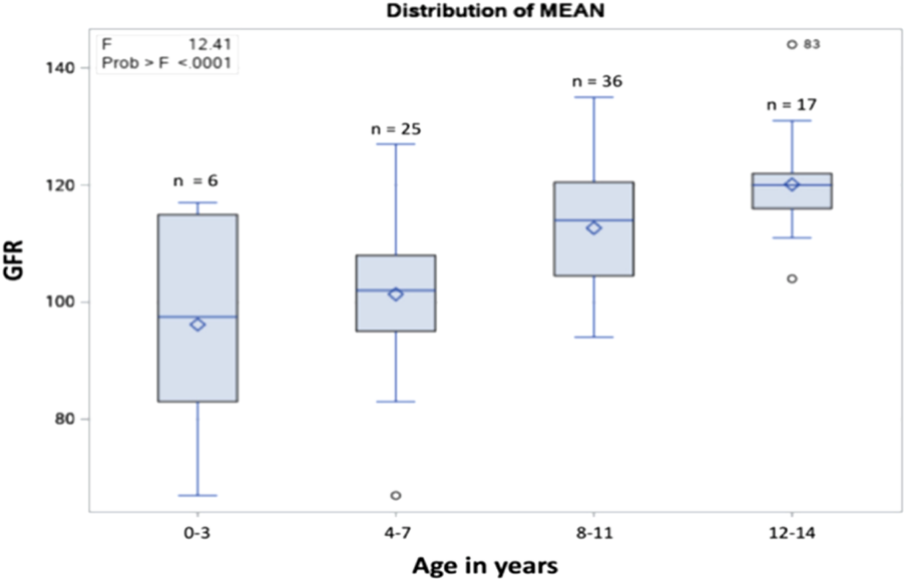
The distribution of the mean GFR per age group.

The most common renal complications were hematuria, proteinuria, glomerular hyperfiltration, hyposthenuria, and GFR dysfunction, observed in 38 (38.4%), 11 (11.1%), 7 (7.1%), and 1 (1.01%) patient, respectively. None experienced acute tubular acidosis or hypertension, as presented in Table 2. Despite evidence of renal complications, only nine (9.09%) patients were referred to the nephrology department. After stratifying the sample into 0–3 years old, 4–7 years old, 8–11 years old, and 12–14 years old age groups, the mean GFR values (ml/min/1.73 m^2^) were 96.16, 101.36, 112.69, and 120.11, respectively, as shown in Figures 1, 2. Among the four age groups, a statistically significant difference (*P* < 0.0001) in the mean GFR was directly proportional to age, as shown in Figure 2.

**Table 2:** Renal complications.

	*N* = 99	%
Hematuria		
Yes	38	38
No	61	62
Proteinuria		
Yes	11	11
No	88	89
Hyposthenuria		
Yes	4	4
No	95	96
Glomerular hyperfiltration		
Yes	7	7
No	92	93
Glomerular dysfunction		
Yes	1	1
No	98	99
Hypertension		
Yes	0	0%
No	99	100%
Radiological changes to the kidney *n* = 92		
Yes	27	29
No	65	71

### Radiological findings

**Figure 3:**
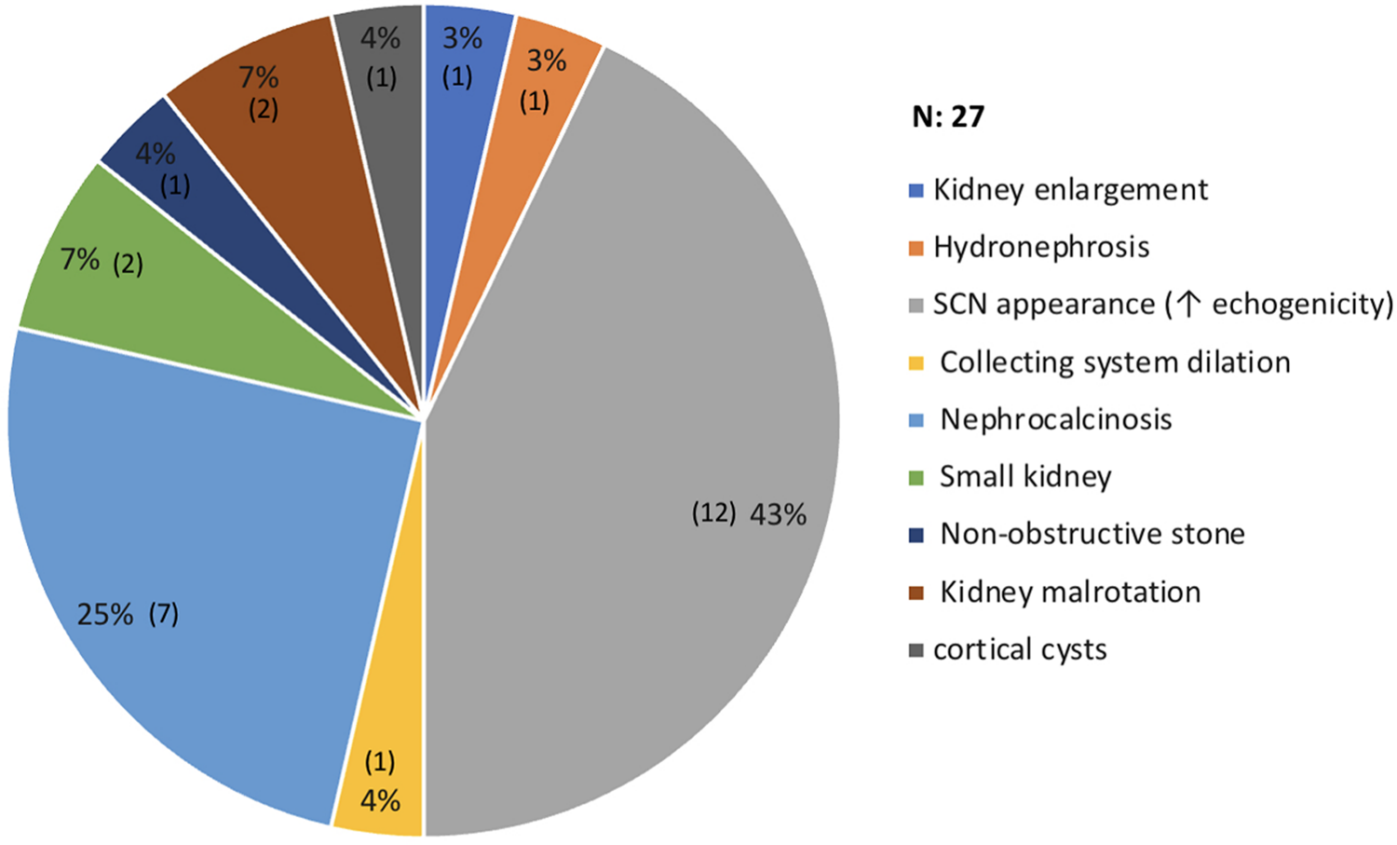
Radiological changes on renal images. *One patient had more than 1 abnormalities.

The most commonly used imaging modality in kidney assessment was ultrasound (US), with 91 (91.91%) of 99 patients having at least one US performed, followed by magnetic resonance imaging (MRI), which was performed in only four patients. After investigating the US results of each patient, the most common findings were increased echogenicity and loss of corticomedullary differentiation, representing a typical SCN appearance 12 (43%), followed by nephrocalcinosis in 7 (25%). On MRI, only one patient had multiple tiny cortical cysts in both kidneys, with no signs of hydronephrosis. Figure 3 summarizes the significant renal imaging findings.

### Disease-modifying agents and the use of nephrotoxic medication

Most patients (92.9%) were administered HU, with a median starting age of 48 (9–168) months and a mean of 58.5 (SD = 33.9) months. When associating the HU initiation age with the number of crises, a statistically significant value (*P* < 0.0017) was noted, correlating earlier HU administration with a decrease in crises. The late age at initiating HU is usually related to late referral or family refusal of early initiation of HU despite counselling. Only five (5.05%) patients were prescribed an iron chelator (deferASIROX).

During inpatient admission, 54 (54.5%) patients received ibuprofen with a mean exposure of 22.6 + 33.4 days, followed by vancomycin which was prescribed to 52 (52.5%) of patients with a mean exposure of to 12.2 + 11.8 days. On the other hand, only one (1.01%) was prescribed aminoglycoside with a mean exposure of 4.5 + 3.5 days. Total exposure to nephrotoxic medication significantly correlated with proteinuria (*P* < 0.04) and an increased risk of UTI (*P* < 0.001).

## Discussion

Sickle cell disease is a multisystem disorder associated with risks of early mortality and morbidity. Because of the nature of the environment in the renal medulla, the red blood cells tend to sickle (21). In this study, we evaluated renal outcomes among pediatric patients with SCD in Saudi Arabia. We found evidence of sickle cell nephropathy (SCN), which manifests at an earlier age as recurrent UTI, hematuria, hyposthenuria, proteinuria, hyperfiltration, glomerular dysfunction, and radiological renal injury. In our study population, different risk factors were associated with SCN development.

Various renal complications contribute to further renal damage in patients with SCD. These include abnormal urinary concentrations, acute kidney injury, hematuria, renal tubular acidosis, hypertension, albuminuria, and hyperfiltration (21). Hematuria was found in 38% of our cohort. Akubuilo et al. demonstrated that 4.1% of their cohort of pediatric patients experienced persistent hematuria, while another published study indicated a prevalence of up to 23% (22). The participants of our study lack consistent follow-up and referral to the nephrology department, which makes the differentiation between persistent and transient hematuria difficult. Albuminuria occurs in 4%–26% of children with SCD. Routine urinalysis primarily detects albumin; however, it is not sensitive enough to detect microalbuminuria, which must be assessed by collecting urine for 24 h (21). Although 11% of our participants had detectable proteinuria on urinalysis, which is consistent with what has been published, none underwent testing for microalbuminuria, making it underdiagnosed in our population. Lopez et al. reported an 11.4% prevalence of hyposthenuria, whereas only 4% of our cohort had low-specific gravity urine. Hyposthenuria can progress to enuresis and polyuria, and usually starts early in infancy, thereby requiring careful monitoring.

Overall, the occurrence of SCN in our study is consistent with that reported in the literature. Two major SCD haplotypes have been documented among Saudi patients: the ArabIndian (AI) and African haplotypes. Previous studies indicate that participants with AI haplotypes typically exhibit a high level of fetal hemoglobin (HbF) and suffer from milder symptoms of SCD, although this has been controversial in most recent studies (23). When comparing the variable HbF levels in our study, no difference in the risk of renal complications was noted. More studies are needed in all populations/geographic regions, which may contribute to a better understanding of phenotypic variability in SCD.

GFR changes with age, and hyperfiltration in SCD occurs early in the disease course, preceding the occurrence of proteinuria. After childhood, GFR begins to decrease in adolescence, and if not prevented by adulthood, it reaches levels indicative of GFR dysfunction and kidney failure. Hirschberg et al. states that in young patients with SCD, the GFR is substantially increased but tends to decrease progressively with time. If this pattern of progression is presumed to be true, it is imperative to determine the causes and risk factors of hyperfiltration to develop early interventions (24). In our study, a statistically significant difference in mean GFR was noted across different age groups. The mean GFR values displayed a proportional relationship with age, suggesting a progressive increase in the hyperfiltration rate, which is consistent with the above findings. This is consistent with previous studies showing that eGFR increases during early childhood, plateaus during adolescence, and decreases as CKD progresses (25, 26).

However, given that this study represented only the pediatric population, and each age group had a different sample size, further studies are required to confirm the pattern of hyperfiltration in childhood progressing to glomerular dysfunction in adulthood.

Our study noted no statistically significant differences between the risk of renal complications and sex. However, boys experienced more renal complications than girls. The sex and role of sex hormones in renal disease are still unclear and require further research, and identifying whether the effects start before, during, or after puberty is challenging. In non-sickle cell settings, many studies support the claim that males have a worse prognosis and accelerated progression of renal diseases when compared to females. This is attributed to different lifestyles, or the damaging effects of testosterone in contrast to the protective effects of estrogens in women (27, 28).

In our cohort, VOC, ACS, and hyperhemolytic crises were among the most frequent crises in SCD, with VOC being the most common cause of hospitalization. The total number of crises per patient was proportional to the risk of proteinuria, hyperfiltration, glomerular dysfunction, and UTI infection. A retrospective study by the University of Alabama at Birmingham reported acute kidney injury (AKI) in 17% of VOC admissions. Moreover, a decrease in hemoglobin levels during pain or ACS events can lead to renal complications from hypoxic-ischemic events, hemolysis, or inflammation (29). HU was the most effective drug for reducing the frequency of painful VOC episodes, ACS, and blood transfusions in patients with SCD by 50% (30). In our study, the use of HU was not associated with renal complications; however, the age at which HU was started demonstrated a statistically significant relationship with the number of crises. Considering this, earlier administration of HU prevents or decreases the number of sickle cell crises in the future, thereby indirectly reducing the prevalence of renal complications in pediatric patients with SCD.

Regarding other risk factors for SCN, the treatment options administered to patients with SCD can have nephrotoxic effects and therapeutic sequelae. Most patients who were simultaneously exposed to vancomycin and ibuprofen displayed a correlation between proteinuria and UTI. A retrospective cohort study reported that 70% of hospitalized children (non-SCD) who were exposed to aminoglycoside for ≥3 days or more than three nephrotoxic medications simultaneously had evidence of residual kidney damage and risk of CKD after six months of exposure. This hints at the increase in risk if SCD were added to the equation (31). Based on our observations, there were poor referrals to the nephrology department despite renal complications. Similarly, underreporting of other symptoms, such as enuresis, and inconsistency in ordering for investigations (urinalysis) were noted. These considerations are crucial since late intervention will most likely negatively affect progression and accelerate irreversible renal damage.

Approximately one-third of our cohort showed renal changes on dedicated renal images, which contradicted the findings described in the literature. Papadaki et al. reported that none of their subjects exhibited renal changes on ultrasonography by 15 years of age (32). A study conducted in Ghana demonstrated the average kidney size and corticomedullary differentiation among children with sickle cell disease under 15 years of age (33).

SCN are a significant complication of SCD. In this study, we assessed the renal outcomes in pediatric patients with SCD. After analyzing the clinical findings of SCN, we concluded that the presence of renal complications in pediatric patients presented a progressive pattern of GFR changes. Identifying and addressing the risk factors for disease progression is vital because early damage invariably occurs during childhood. A distinct pediatric SCN staging method is required to effectively identify intervention options and slow the progression of renal dysfunction.

## Study limitations and future direction

Our study had some limitations. First, the results may not be completely generalizable because of the small sample size and single-center nature of the study. Second, our retrospective case study resulted in insufficient, repetitive, and imprecise documentation. Finally, the lack of regional studies in Saudi Arabia on the topic precludes a comparison of our population with a similar group of patients. Considering these limitations, we postulate that a prospective, multicenter cohort study with a more representative sample will enhance our understanding of disease progression, thereby helping physicians establish a systemic management plan. An essential recommendation for physicians is early and routine screening of renal function in SCD, which is a key factor for early intervention and prevention of potentially irreversible renal outcomes.

## Data Availability

The raw data supporting the conclusions of this article will be made available by the authors, without undue reservation.
